# Exosome inhibition improves response to first‐line therapy in small cell lung cancer

**DOI:** 10.1111/jcmm.18138

**Published:** 2024-02-14

**Authors:** Nesrin Irep, Kubilay Inci, Pervin Elvan Tokgun, Onur Tokgun

**Affiliations:** ^1^ Department of Cancer Molecular Biology, Institution of Health Sciences Pamukkale University Denizli Turkey; ^2^ Department of Medical Genetics, Faculty of Medicine Pamukkale University Denizli Turkey

**Keywords:** combination therapy, exosome, nsMase2, RAB27A, SCLC

## Abstract

Exosomes are recognized as important mediators of cell‐to‐cell communication, facilitating carcinogenesis. Although there have been significant advancements in exosome research in recent decades, no drugs that target the inhibition of sEV secretion have been approved for human use. For this study, we employed GW4869 and Nexinhib20 as inhibitors of exosome synthesis and trafficking combined. First, we found that Nexinhib20 and GW4869 effectively inhibited RAB27A and neutral sphingomyelinase 2 (nSMase2) nsMase2. Interestingly, the inhibition of nsMase2 and RAB27A decreased expression of CD9, CD63 and Tsg101, both at RNA and protein levels. We used a combination treatment strategy of cisplatin/etoposide plus GW4869 or Nexinhib20 on small cell lung cancer (SCLC) cell lines. The combination treatment of GW4869 or Nexinhib20 effectively enhanced the inhibitory effects of first‐line chemotherapy on the SCLC cells. Furthermore, we demonstrated that reducing exosome release through GW4869 and Nexinhib20 treatment effectively reduced cellular proliferation and significantly induced apoptosis in SCLC cells. Also, we showed that combining exosome inhibition with chemotherapy has a significant synergistic effect on cellular proliferation. We also found increased p53 and p21 expressions with western blot and significantly changing Bax, BCL2, caspase‐3 and caspase‐9 expressions. Inhibiting the exosome pathway offers opportunities for developing novel, effective treatment strategies for SCLC.

## 1 INTRODUCTION

Small cell lung cancer (SCLC) is among the most aggressive types of lung cancer, known for its rapid growth, quick spread, and high mortality rate.^1^ SCLC accounts for approximately 15% of all lung cancer diagnoses, and platinum‐etoposide chemotherapy is the cornerstone of recommended treatment for SCLC.^2^ Etoposide plus cisplatin (EP) is the standard regimen for chemotherapy in patients with SCLC, along with early, concurrent thoracic radiation therapy (RT).^3^, ^4^, ^5^ Even after more than three decades of conducting Phase III clinical trials, the 5‐year survival rate for SCLC stands at 6%, and these unsatisfactory outcomes are not due to a lack of effort.^3^ Unfortunately, poor outcomes are mainly due to a lack of effective therapies to combat both limited and extensive‐stage SCLC.^2^ To further the development of effective treatments, there is a need for new insights into the biology of SCLC.

Exosomes are one of the sub‐categories of small extracellular vesicles (EVs), which have a nano‐vesicular architecture with a diameter of around 30–150 nm. They are found in many cells and are released by the fusion of multivesicular bodies (MVBs) with the plasma membrane.^6^ In exosomes, encapsulating various biomolecules, including lipids, proteins, coding and non‐coding RNAs, and DNA species, promotes the survival and dissemination of cancer cells against stress, chemoresistance and immune surveillance.^7^, ^8^, ^9^, ^10^ Tumour‐derived exosomes have a critical role in the tumour environment through short‐ and long‐distance delivery of biological molecules.^10^, ^11^, ^12^, ^13^ Increasing evidence has shown that exosomes derived from tumour cells affect the proliferation and metastasis of recipient tumour cells.^14^, ^15^, ^16^ At the same time, they suppress T‐cell proliferation and interfere with CD8 T‐cell activation, which contributes to immune evasion.^17^, ^18^ Therefore, preventing the formation and release of exosomes for various cancers has significant potential to establish successful treatment methods in the future.

Given the focus on exosome synthesis and release mechanisms, techniques for targeting exosome inhibition have been developed. Genetic manipulation and pharmacological inhibitors are the most widely studied approaches.^17^, ^18^, ^19^ For example, Greenberg et al.^20^ reported that ketoconazole enhanced the efficacy of sunitinib by inhibiting exosomes in sunitinib‐resistant 786‐O cell lines. Im et al. have shown that sulfisoxazole enhances the efficacy of docetaxel in the progression of breast cancer by inhibiting the production of exosomes.^21^ However, it is widely recognized that such studies in small‐cell lung cancer are lacking. Although evidence for the critical role of exosomal communication in small‐cell lung cancer is rapidly accumulating, there has been little progress in identifying therapeutic candidates to prevent exosomal secretion. In addition, platinum‐based etoposide treatment is widely used in small‐cell lung cancer and provides a high early success rate. However, patient survival and treatment efficacy are relatively poor due to the rapid development of relapse. Therefore, we researched to investigate the effects of inhibiting the biogenesis/secretion of exosomes using specific inhibitors on SCLC and its contribution to conventional therapy. We have found that inhibiting exosome biogenesis effectively decreases the proliferative activity of cancer cells and increases apoptosis in small‐cell lung cancer. Moreover, combining exosome pathway inhibition with platinum‐based etoposide therapy has improved first‐line therapy's efficacy on small‐cell lung cancer cells.

## 2 MATERIALS AND METHODS

### 2.1 Cell culture, preparation of stock inhibitor solution including cisplatin, etoposide, GW4869 and Nexinhib20

The cell lines H889 and H524 were obtained from the American Type Culture Collection (ATCC), and N417 was obtained from Dr. Jun Yokota. H889, H524 and N417 SCLC cell lines were cultured in RPMI‐1640 media with 10% foetal bovine serum (FBS) and 1% Penisilin/Streptomycin (Gibco, USA). All cells were grown in 5% CO_2_ humidified air at 37°C. In all experiments, the viability of the SCLC cells was 95%. Cisplatin (Cat. no. 13119) was obtained from Cayman Chemical (Canada). Etoposide (Cat. no. A10373) and GW4869 (Cat. no. A11974) were purchased from Adooq Bioscience (USA). Nexinhib20 (Cat. no. SML1919) was purchased from Sigma Aldrich. GW4869, Etoposide, and Nexinhib20 stock solutions were prepared in DMSO, while cisplatin was prepared in distilled water. Fresh batches of inhibitors are prepared for each experiment. The referred abbreviations of experimental groups are as follows: cisplatin (Cis), etoposide (Eto), GW4869 (GW) Nexinhib20 (Nex), cisplatin plus etoposide (Cis + Eto), cisplatin plus etoposide combined with GW4869 (Cis + Eto + GW) and cisplatin plus etoposide combined with Nexinhib20 (Cis + Eto + Nex).

### 2.2 Cytotoxicity assay

We measured the dose‐dependent effects of cisplatin, etoposide, GW4869 and Nexinhib20 by using the MTT [3‐ (4,5‐dimethylthiazol‐2‐yl)−2,5‐diphenyltetrazolium bromide] assay (ROCHE Cell Proliferation Kit I, MTT, Cat. no. 11465007001). N417, H524 and H889 cells were seeded at a density of 1 × 10^4^ cells/well in a 96‐well plate. We treated the cells with varying concentrations of cisplatin (0, 10, 50, 100, 150 and 200 μM), etoposide (0, 1, 5, 10, 25, 50 and 100 μM), GW4869 (0, 1, 10, 20 and 50 μM) and Nexinhib20 (Nex) (0, 1, 2, 5, 7.5, 10 and 20 μM) at 37°C for 48 h. We also analysed the cytotoxicity of DMSO (0.5%), which is used to dissolve inhibitors. At the end of the incubation, we followed the protocol of ROCHE Cell Proliferation Kit I. Using a microplate reader; we measured the optical density of each well at 560 nm. We used the GraphPad Prism.8.0.1 to calculate the IC_50_ dosage of cisplatin, etoposide, GW4869 and Nexinhib20.

### 2.3 Exosome isolation, identification with Transmission electron microscopy, and quantification with EXOCET assay

SCLC cell lines were harvested once the cell confluence reached 80%–90%. Following the removal of the FBS‐containing cell medium, H889, H524 and N417 cells were placed in exosome‐depleted FBS‐containing RPMI‐1640 medium at a density of 5 × 10^5^ cells per well in a six‐well plate. IC_50_ values of each molecule performed all treatments for each cell line. For the combined treatments with exosome inhibitors and first‐line chemotherapy, SCLC cells were treated with the IC_50_ value for Nexinhib20 or GW4869 and combined with IC_50_ concentrations of cisplatin and etoposide for 48 h. At the end of the treatment period, the media were pooled for quantitative and qualitative analysis, and cells were counted using the RWD C100‐SE automated cell counter with trypan blue. For the exosome counting, the cell medium was centrifuged at 2000× g for 30 min to concentrate exosomes and filtered through a 0.22 μm filter (Millipore, USA). Samples were incubated with 0.5 volumes of exosome isolation reagent (Qiagen, miRCURY Exosome Cell/Urine/CSF Kit Cat. no. /ID: 76743, USA) at 4°C overnight and were centrifuged at 10,000 g for 1 h at 4°C. Finally, the exosome pellets were resuspended in 100 μL PBS and stored at −80°C for later use. Transmission electron microscopy (TEM) was performed at the Pamukkale University Advanced Research Laboratory Center. TEM analysis was performed as previously described by Tokgun et al.^22^, ^23^ According to the manufacturer's protocol, the amount of exosomes derived from SCLC cell lines was quantified by EXOCET assay (System Biosciences, USA). Exosome/cell number values were normalized to the control sample of cell numbers and exosome amounts obtained at 48 h.

### 2.4 qRT‐PCR analysis

Total RNA was extracted from all groups of SCLC cells using the TRIzol LS Reagent (Invitrogen, USA) and RNeasy Mini Kit (Qiagen, Germany), according to the manufacturer's instructions. Reverse transcription was performed following the instructions of the cDNA reverse transcription kit (OneScript® Plus cDNA Synthesis Kit). β‐actin was used as the endogenous control, and the relative transcription levels of target genes were calculated by the 2^−ΔΔCt^ method compared to control groups. All primers used in this study were synthesized by Sentebiolab (Ankara, Turkey) and the mRNA primer pairs used in the study are as follows: β‐actin (forward, 5′‐CGCGAGAAGATGACCCAGGA‐3′; reverse, 5′‐GATAGCACAGCCTGGATAGCAAC‐3′), CDK‐6 (forward, 5′‐TTGTGACAGACATCGACGAG‐3′; reverse, 5′‐GACAGGTGAGAATGCAGGTT‐3′), Cylin‐D1 (forward, 5′‐CAGACCAGCCTAACAGATTTC‐3′; reverse, 5′‐TGACCCACAGCAGAAGAAG‐3′), CD9 (forward, 5′‐GGGGGCGTGGAACAGTTTAT‐3; reverse, 5′‐GCGCCGATGATGTGGAATTT‐3′), CD63 (forward, 5′‐ ATGATCACGTTTGCCATCTT‐3′; reverse, 5′‐AGGGATTTTCTCCAATCTG‐3′) TSG101 (forward, 5′‐CTCTCATCTCTGCGGTCAGT‐3′; reverse, 5′‐TCAACCTCGGCTACTTCTTG‐3′), Rab27a (forward, 5′‐GTTGATGGAGCGAACTGCTT‐3′; reverse, 5′‐CTACGAAACCTCTCCTGCCC‐3′), nsMase2 (forward, 5′‐ GAAGCACACCTCAGGACCAAAG‐3′; reverse, 5′‐CAGCCAGTCCTGAAGCAGGTC‐3′), Bcl‐2 forward, 5′‐CTGCACCTGACGCCCTTCACC‐3′; reverse, 5′‐ CACATGACCCCACCGAACTCAAAGA‐3′, Casp9 forward, 5′‐GTTTGAGGACCTTCGACCAG‐3′; reverse, 5′‐ CAACGTACCAAGGAGCCACTC‐3′, Casp3 forward, 5′‐GGAAGCGAATCAATGGACTC‐3′; reverse, 5′‐ GCATCGACATCTGTACCAGA‐3′, Bax forward, 5′‐TCAGGATGCGTCCACCAAGA‐3′; reverse, 5′‐ TGTGTCCACGGCGGCAATCA‐3′.

### 2.5 Western blotting

Protein was isolated from all cell groups using RIPA lysis reagent (Santa Cruz, USA) supplemented with protease (Roche) and phosphatase (Sigma) inhibitor cocktails after 48 h treatment with the indicated treatment strategy. The quantity was measured using the Bradford assay. The 60 μg/well protein was separated using 10% SDS PAGE gels and transferred onto the polyvinylidene fluoride (PVDF) membrane using the Trans‐Blot Turbo transfer system (Bio‐Rad, USA). The membrane was blocked with 5% nonfat milk at room temperature for 1 h and incubated with primary antibodies as follows: anti‐human antibody mouse nsMase2 (G‐6, sc‐166637, 1:2000), mouse TSG101 (C‐2, sc‐7964, 1:1000), mouse RAB27A (E‐8, sc‐74586, 1:500), rabbit CD9 (D3H4P, 1:2500), rabbit Tubulin (9F3, 2128, 1:5000), rabbit GAPDH (D16H11, 5174, 1:5000), mouse p53 (Dako M7001, 1:2000), p21 (F‐5, sc‐6246) at 4°C overnight. Membranes underwent TBST washing and were then incubated with anti‐rabbit secondary (Cell Signaling, 7074, 1:1000) and anti‐mouse secondary (Cell Signaling, 7076, 1:1000) antibodies. The Odyssey® Fc Imaging System (LI‐COR Biosciences) captured the protein band images. Quantitative analysis of target protein bands calculated according to the grey value ratio of the target band to GAPDH or tubulin bands.

### 2.6 Cell proliferation assay

As previously reported,^24^ we conducted a cell proliferation assay to investigate the impact of exosome pathway inhibition on cell proliferation in SCLC cell lines. A total of 5 × 10^5^ cells were seeded in RPMI 1640 medium, supplemented with exosome‐depleted FBS (Gibco, USA), in each well of a 6‐well plate, and then treated with drug groups, including GW, Nex, cisplatin‐plus etoposide and combinations such as GW + Cis + Eto and Nex + Cis + eto, for 48 h. Fold changes in SCLC cell groups after 48 h of exposure to the specified treatment strategies were calculated using untreated groups as controls.

### 2.7 TUNEL analysis

H889, H524, and N417 cells (3×10^4^ cells/well) were seeded into 24‐well plates. The cells were treated with various drug combinations, including GW, Nex, Cis + Eto, Cis + Eto + GW and Cis + Eto + Nex, for 24 h. Subsequently, a TUNEL assay was conducted using the Roche In Situ Cell Death Detection Kit protocol, AP (11684809910, Version 12, Germany). After 24 h of exposure to the designated treatment strategy, the cells were collected, centrifuged, and the supernatants were removed. After fixing with 4% paraformaldehyde for 1 h, the cells underwent treatment with permeabilization buffer (0.1% Triton X‐100 in 0.1% sodium citrate) at 4°C for 2 min. Following a wash with PBS solution, the cells were incubated at 37°C in the dark for 60 min with 50 μL fluorescent label solution consisting of 5 μL of terminal deoxynucleotidyl transferase (TdT) and 45 μL of fluorescein‐labelled 20‐deoxyuridine 50‐triphosphate (dUTP) solution per sample. Three random fields were chosen from each section to enumerate the number of positive cells, and green TUNEL‐positive cells were observed under a fluorescent microscope. We calculated the fold change of green‐positive cells by comparing them with their non‐treated counterparts.

### 2.8 Statistical analysis

All data analysis was performed using the SPSS 21.0 software (IBM, USA) and the Graphpad Prism 8.0.1 program. The data were reported as mean ± SEM from at least three biological replicates. Unpaired Student's *t*‐test was used to compare the data between the treated and control groups. One‐way analysis of variance (ANOVA) was used to compare two or more groups. The standard deviation (SD) was represented by error bars. *p < 0.05, **p < 0.01, ***p < 0.001, ****p < 0.0001.

## 3 RESULTS

### 3.1 Inhibitory effects of cisplatin, etoposide, GW4869 and Nexinhib20 on SCLC cell lines in vitro

**FIGURE 1:**
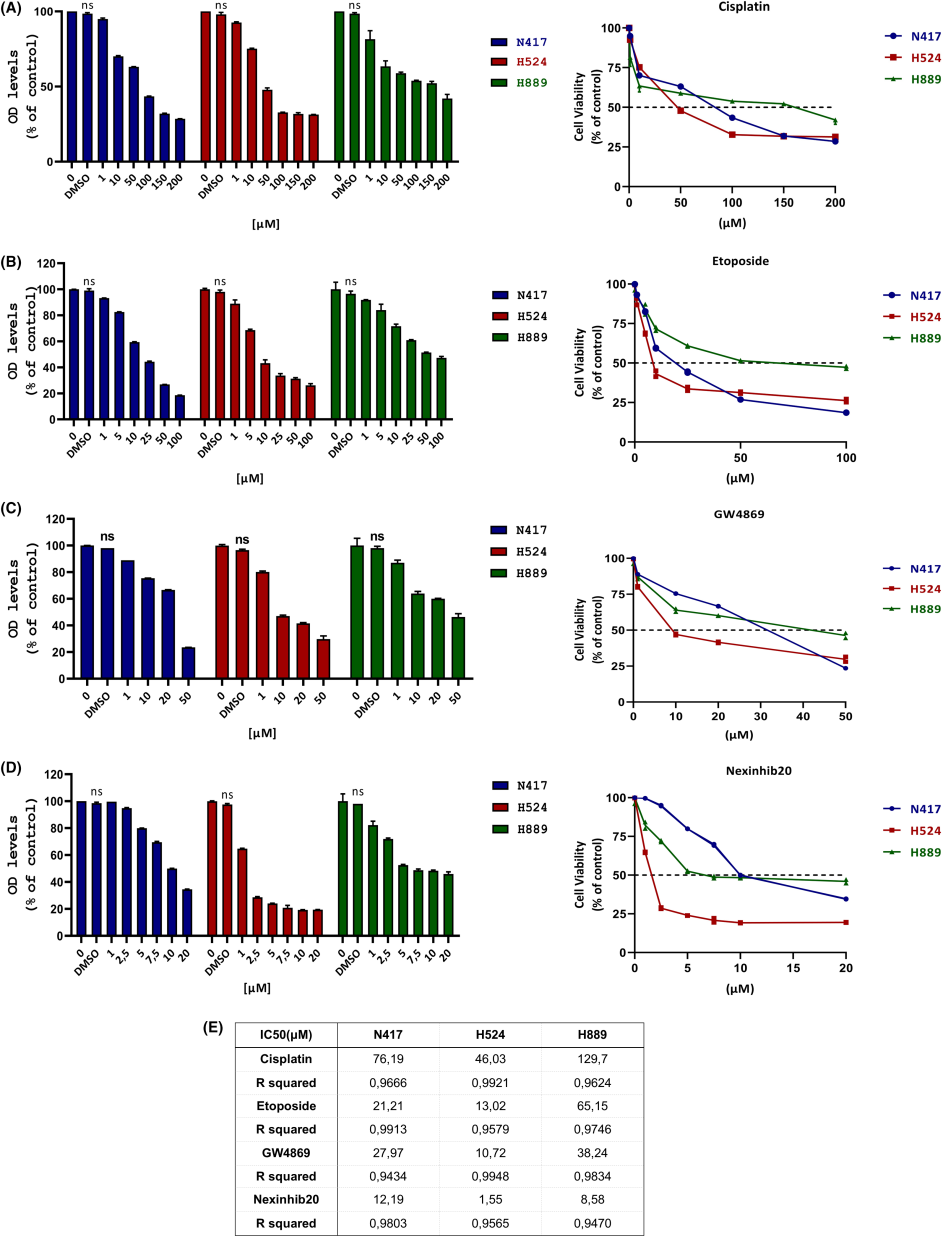
The cytotoxic effects of cisplatin, etoposide, GW4869, and Nexinhib20 at different concentrations on the growth of small cell lung cancer cell lines. H889, H524 and N417 SCLC cell lines were treated for 48 h with cisplatin at different concentrations (0, 1, 10, 50, 100, 150 and 200 μM) (A); etoposide at different concentrations (0, 1, 5, 10, 25, 50 and 100 μM) (B); GW4869 at different concentrations (0, 1, 10, 20 and 50 μM) (C); Nexinhib20 at different concentrations (0, 1, 2.5, 5, 7.5, 10 and 20 μM) (D). (E) The IC50 concentrations of cisplatin, etoposide, GW4869 and Nexinhib20 are shown. Statistically, the untreated groups for each cell line were estimated as 100%. A represents the mean ± SD values obtained from three independent replicates (*n* = 3). MTT assay was used to determine cell viability of all cell line curves.

The initial step was to utilize the MTT assay to examine the dosage‐dependent outcomes of cisplatin, etoposide, GW4869 and Nexinhib20 in the SCLC cell lines, followed by calculating IC50 doses of drugs. It was found in Figure 1A–D that N417, H524 and H889 cells experienced a decline in survival that was dependent on the dosage when treated with cisplatin at concentrations ranging from 1 to 200 μM, etoposide from 1 to 100 μM, GW4869 from 1 to 50 μM and Nexinhib20 from 1 to 20 μM for 48 h in comparison to control cells. The IC50 values for N417, H524 and H889 cells, depending on the concentration of cisplatin, etoposide, GW4869 and Nexinhib20 treatments, are presented in Figure 1E. H524 cells required lower IC50 concentrations of cisplatin, etoposide, GW4869 and Nexinhib20 than N417 and H889 cells (Figure 1E).

### 3.2 GW4869 and Nexinhib20 decreased expressions of RAB27A, nsMase2, CD9, CD63 and TSG101 genes at both RNA and protein levels quantitatively and inhibited exosome production and trafficking in SCLC cells effectively

**FIGURE 2:**
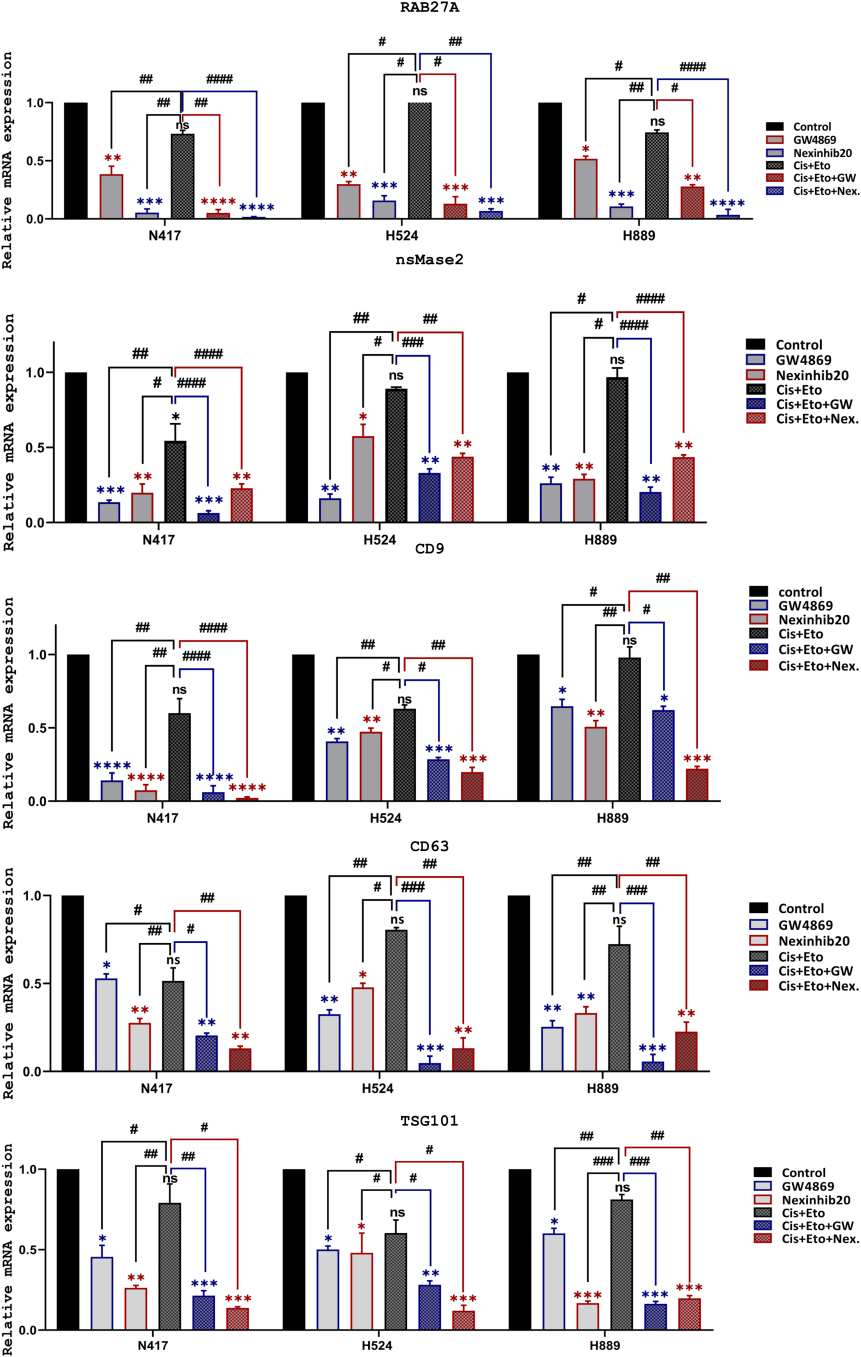
GW4869 and Nexinhib20 quantitatively reduced the RNA expression of exosomal marker genes in small cell lung cancer cells. qRT‐PCR analysis revealed a reduction of exosomal biomarker genes, including RAB27A, nsMase2, TSG101, CD9, and CD63 genes at the RNA level in SCLC cells following treatment with the IC50 dose of GW4869 or Nexinhib20 in both alone and combination Cis + Eto therapy for 48 h. The significance levels of expression changes in exosome marker genes due to alone and combined treatment of inhibitors were evaluated compared to both the control and Cis + Eto treated groups. Experiments were performed with 95% confluent cells, and the data shown are mean values ± SD derived from three independent experiments. Significances were determined by comparison to untreated control group cells using an unpaired two‐tailed Student's *t*‐test. Different punctuation marks have been used for *p*‐value. The significance of the data between control vs. all treatments was shown using an asterisk (*), whereas the comparison between Cis + Eto versus all treatments was indicated as hash (^#^). * p < 0.05, **p < 0.01, ***p < 0.001, ****p < 0.0001, ^#^ *p* < 0.05, ^##^ *p* < 0.01, and ^###^ *p* < 0.001.

**FIGURE 3:**
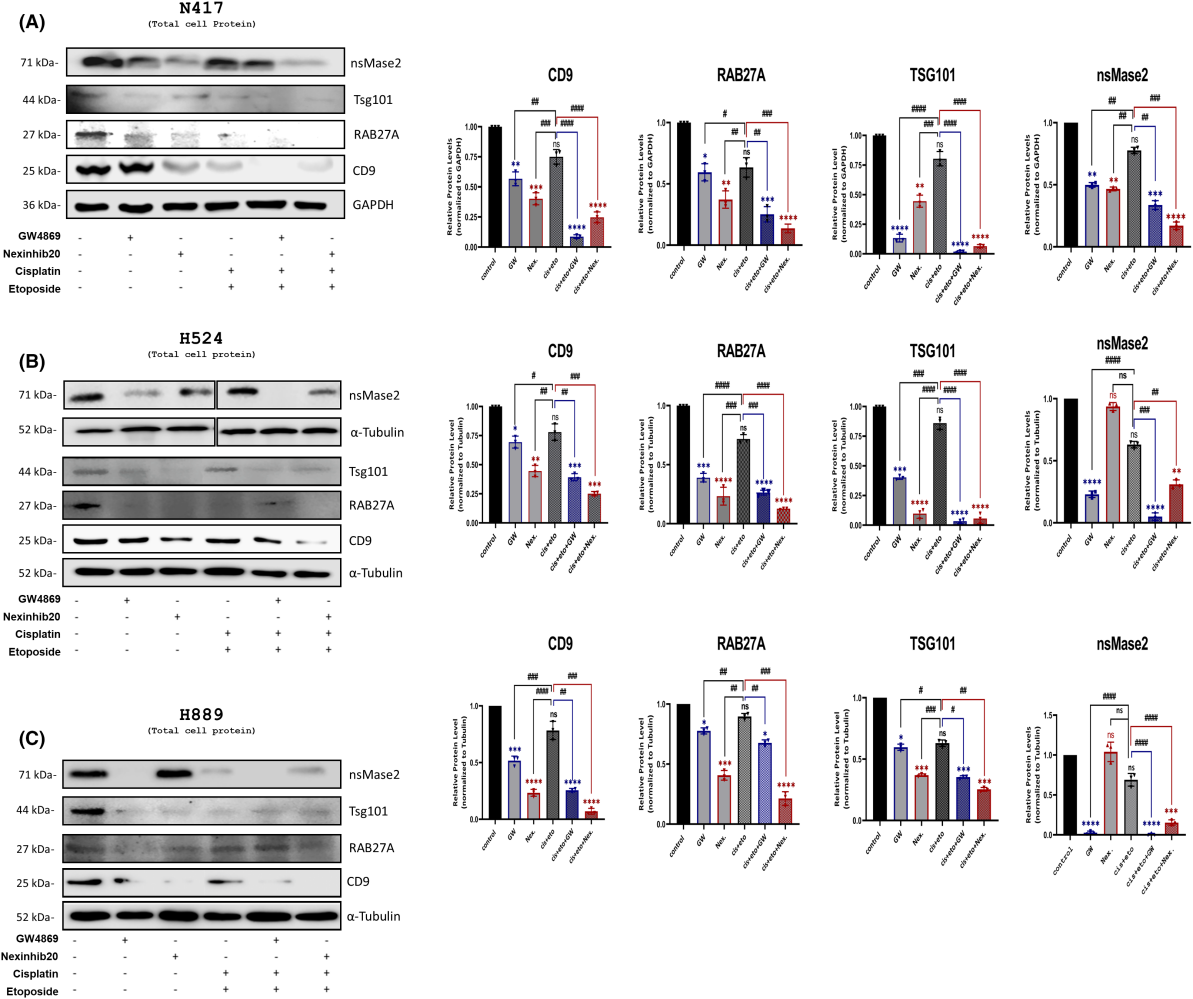
GW4869 and Nexinhib20, alone and in combination with Cis + Eto, dramatically reduced the protein levels of genes responsible for critical functions in exosome biogenesis. Protein levels of nsMase2 (71 kDa), TSG101 (44 kDa), RAB27A (27 kDa), CD9 (25 kDa), and GAPDH (36 kDa) (which served as the control gene for N417 cells), as well as α‐tubulin (52 kDa) (which served as the control gene for H524 and H889), were identified using Western blot analysis in N417 (A), H524 (B), and H889 (C) cells, and protein bands were quantified using Image Studio software compared to untreated controls are shown as column graphs on the left. The significance levels of expression changes in exosome marker genes due to the alone and combined treatment of inhibitors were evaluated and compared to both the control and Cis + Eto treated groups. Significances were determined by comparison to untreated control group cells using an unpaired two‐tailed Student's *t*‐test. Different punctuation marks have been used for the *p*‐value. The significance of the data between control vs. all treatments was shown using an asterisk (*), whereas the comparison between Cis + Eto versus all treatments was indicated as hash (^#^). * p < 0.05, **p < 0.01, ***p < 0.001, ****p < 0.0001, ^#^ *p* < 0.05, ^##^ *p* < 0.01, and ^###^ *p* < 0.001.

GW4869 is an inhibitor of nsMase2 (neutral sphingomyelinase), an enzyme that forms vesicles related to exosome production that does not depend on the ESCRT pathway.^25^ In contrast, Nexinhib20 impedes Rab27a, a small GTPase that is a vital regulator of exosomal trafficking within cells.^26^ In light of this, we employed two inhibitors in our research that target different aspects involved in exosome formation. We cultivated N417, H524, and H889 cells in six‐well dishes until they achieved roughly 80% confluency. We then divided cells into six groups: untreated (control), GW4869, Nexinhib20, cisplatin+etoposide (Cis + Eto), Cis + Eto + GW4869 (GW) and cis + eto + Nexinhib20 (Nex). We examined the expressions of significant exosome biogenesis regulator genes after treatment with GW4869 and Nexinhib20, both separately and in combination. Exosomal markers, RAB27A, nsMase2, CD9, CD63 and TSG101, were analysed.^27^, ^28^ The evaluation of exosomal marker gene expressions was performed using qRT‐PCR. In SCLC cells, the expressions of RAB27A and nsMase2, CD63, CD9 and TSG101 were significantly reduced in all treatment groups when compared to the control (*p < 0.05, **p < 0.01, ***p < 0.001, ****p < 0.0001) (Figure 2). Based on the qRT‐PCR results, Western blot analysis showed a statistically significant reduction in protein expression of CD9, RAB27A, TSG101 and nsMase2 in the GW4869 and Nexinhib20‐treated groups (as shown in Figure 3A–C, in N417, H524 and H889, respectively; **p < 0.01, ***p < 0.001 and ****p < 0.0001). In addition, the expression of exosome marker genes was significantly reduced at both protein and RNA levels when the exosome inhibitors Nex and GW were combined with first‐line chemotherapy independently (^#^ *p* < 0.05, ^##^ *p* < 0.01 and ^###^ *p* < 0.001).

**FIGURE 4:**
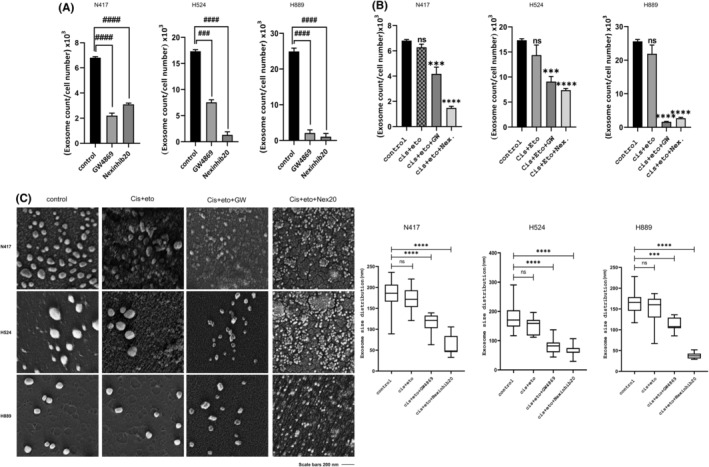
EXOCET analysis and TEM demonstrated quantitative and qualitative changes in the characteristics of exosomes released from SCLC cells within 48 h of exposure to the GW4869 and Nexinhib20, alone and in combination with Cis + Eto. The results of EXOCET quantification analysis showed a significant decrease in the number of exosomes secreted from SCLC cells (N417, H524 and H889) treated with GW and Nex separately. (A) Exosome number changes as a result of combination therapy. (B) Representative TEM images of isolated exosomes on the left. Exosome size distribution of SCLC cells depending on combination therapy. Statistical differences in all groups of N417, H524 and H889 cells compared to non‐treated control counterparts and analysed with GraphPad Prism 8.0.1. (C) Data were analysed using the Student's *t*‐test with significant differences having a *p < 0.05, **p < 0.01, ***p < 0.001, ****p < 0.0001 and ^###^ *p* < 0.001 and ^####^ *p* < 0.0001.

In current work, treatment with the IC_50_ doses of GW4869 and Nexinhib20 for 48 h before exosome isolation from SCLC cell lines resulted in a substantial decrease in total exosome release compared to the control groups (Figure 4A) (^###^ *p* < 0.001 and ^####^ *p* < 0.0001). Moreover, exosome production was considerably reduced following the treatment of Cis + Eto therapy with GW4869 or Nexinhib20 independently (Figure 4B) (***p < 0.001 and ****p < 0.0001). Consistent with the exosome count results, the size of exosomes was qualitatively reduced after exosome release was suppressed with GW and Nex in addition to Cis + Eto therapy (Figure 4C) (***p < 0.001 and ****p < 0.0001). However, the results of the exosome count and exosome size analysis showed that Cis + Eto treatment did not affect the number and size of exosomes (Figure 4B,C). As a result, the data we obtained show that the change in exosome levels is due to the inhibition of exosome release, independent of the change in cell survival caused by the inhibitors.

### 3.3 Co‐administration of GW4869 or Nexinhib20 with first‐line therapy enhanced the inhibitory effects of first‐line therapy on SCLC cell viability

**FIGURE 5:**
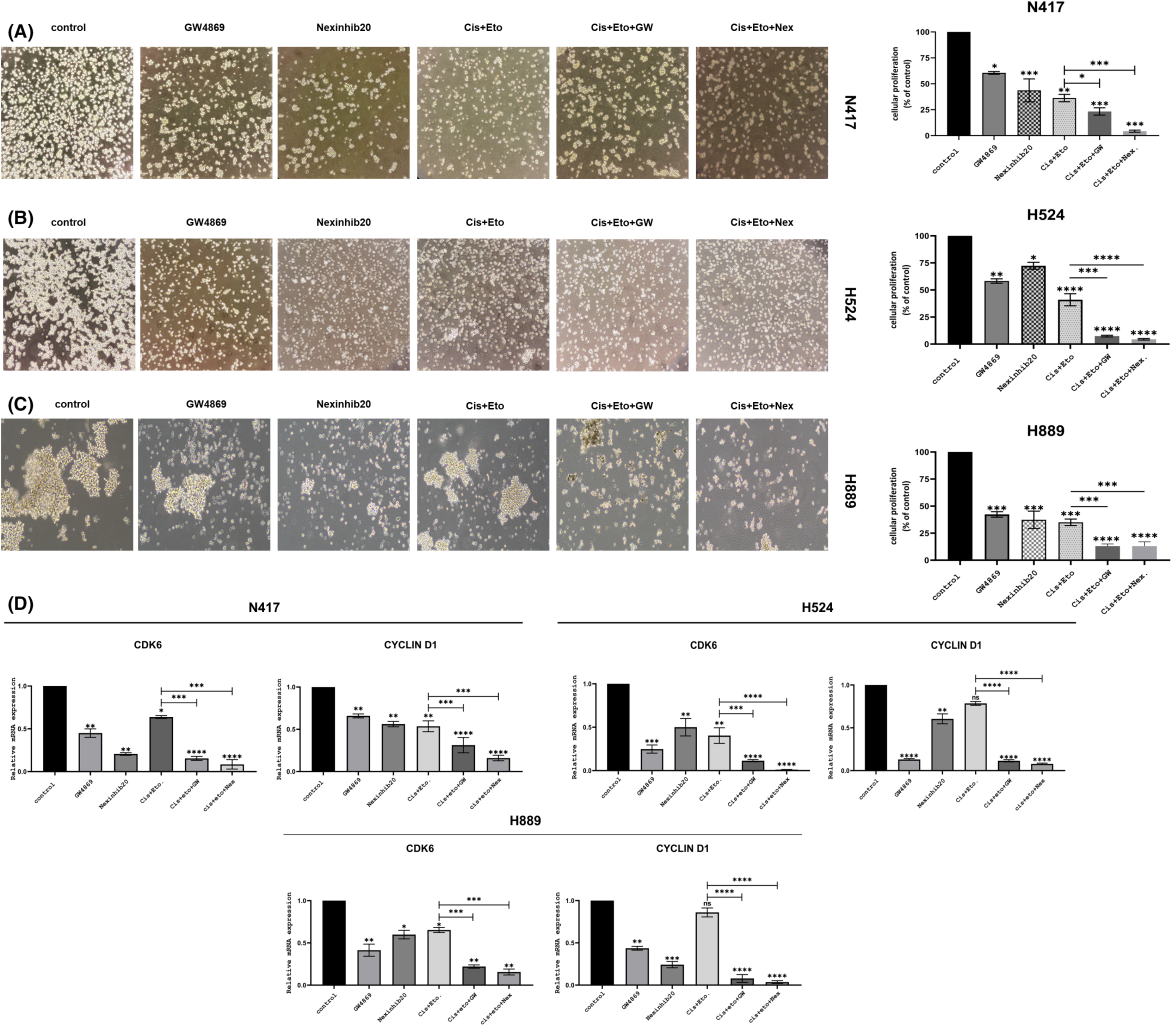
Exosome synthesis in SCLC cells was inhibited using the GW4869 and Nexinhib20 inhibitors, which enhanced the inhibitory impact of Cis + Eto on cell growth. The proliferation activity of N417 (A), H524 (B) and H889 (C) cell groups treated with GW4869, Nexinhib20, Cis + Eto, Cis + Eto + GW and Cis + Eto + Nex was evaluated using the MTT assay (on the right). Representative images (original magnification, ×20) of N417(A), H524 (B) and H889 (C) cells were treated with IC50 dose of GW4869 or nexinhib20 in both alone and combination Cis + Eto therapy for 48 h (on the left). Cyclin D1 and CDK6 mRNA levels were examined using qRT‐PCR in N417, H524 and H889 cells treated with IC50 dose of GW4869 or Nexinhib20 in both alone and combination Cis + Eto therapy for 48 h (D). The mean ± SD (error bars) shows quantitative data from three independent experiments. *p < 0.05, **p < 0.01, ***p < 0.001, ****p < 0.0001 (unpaired Student's *t*‐test, one‐way ANOVA test).

To investigate the combinatorial impact of exosome pathway inhibition on SCLC cell lines, we utilized the MTT assay previously employed in Tokgun et al.^13^, ^24^ studies. We used combinations of GW4869 and Nexinhib20 to evaluate the impact of exosome pathway inhibitors in enhancing the efficacy of cisplatin plus etoposide‐based first‐line therapy. Upon exosome pathway inhibition, we observed a significant improvement in the growth inhibitory effect of Cis + Eto on the proliferation of SCLC cells (Figure 5). The proliferation rate of the inhibitor combination with Cis + Eto was 22.96% for GW4869 and 3.64% for Nexinhib20 in N417 cells, as shown in Figure 5A. In H524 cells, the proliferation rate of Nexinhib20 was 4.66%, while that of GW4869 was 6.53%, as shown in Figure 5B, and in H889 cells, the proliferation rate of the combination of inhibitors with Cis + Eto was 13% for GW4869 and 13.1% for Nexinhib20, as shown in Figure 5C. Thus, our findings indicate that inhibiting exosomal pathways with either GW4869 or nexinhib20 increases the effectiveness of first‐line therapy in inhibiting the proliferation of SCLC cells.

Treatment of H889, H524 and N417 cells with GW4869 and Nexinhib20 for 48 h reduced RNA levels of the cell cycle regulatory genes CDK6 and cyclin D1 (Figure 5D) (*p* < 0.01 for N417; *p* < 0.001, *p* < 0.0001 for H524; *p* < 0.01 and *p* < 0.05 for H889). In addition, the RNA expression levels of cyclin D1 and CDK6 were significantly lower in the SCLC cell groups receiving first‐line therapy together with exosome pathway inhibitors than in the SCLC cell groups receiving cisplatin‐plus‐etoposide treatment and untreated groups (Figure 5D).

Inhibition of exosomal pathways reduced proliferation and improved the efficacy of cisplatin plus etoposide treatment in SCLC cells, including H889, H524 and N417 cells, which exhibit various characteristics of small‐cell lung cancer.

### 3.4 The inhibition of exosomes through GW4869 and Nexinhib20 enhanced first‐line therapy‐mediated apoptosis in SCLC cells

**FIGURE 6:**
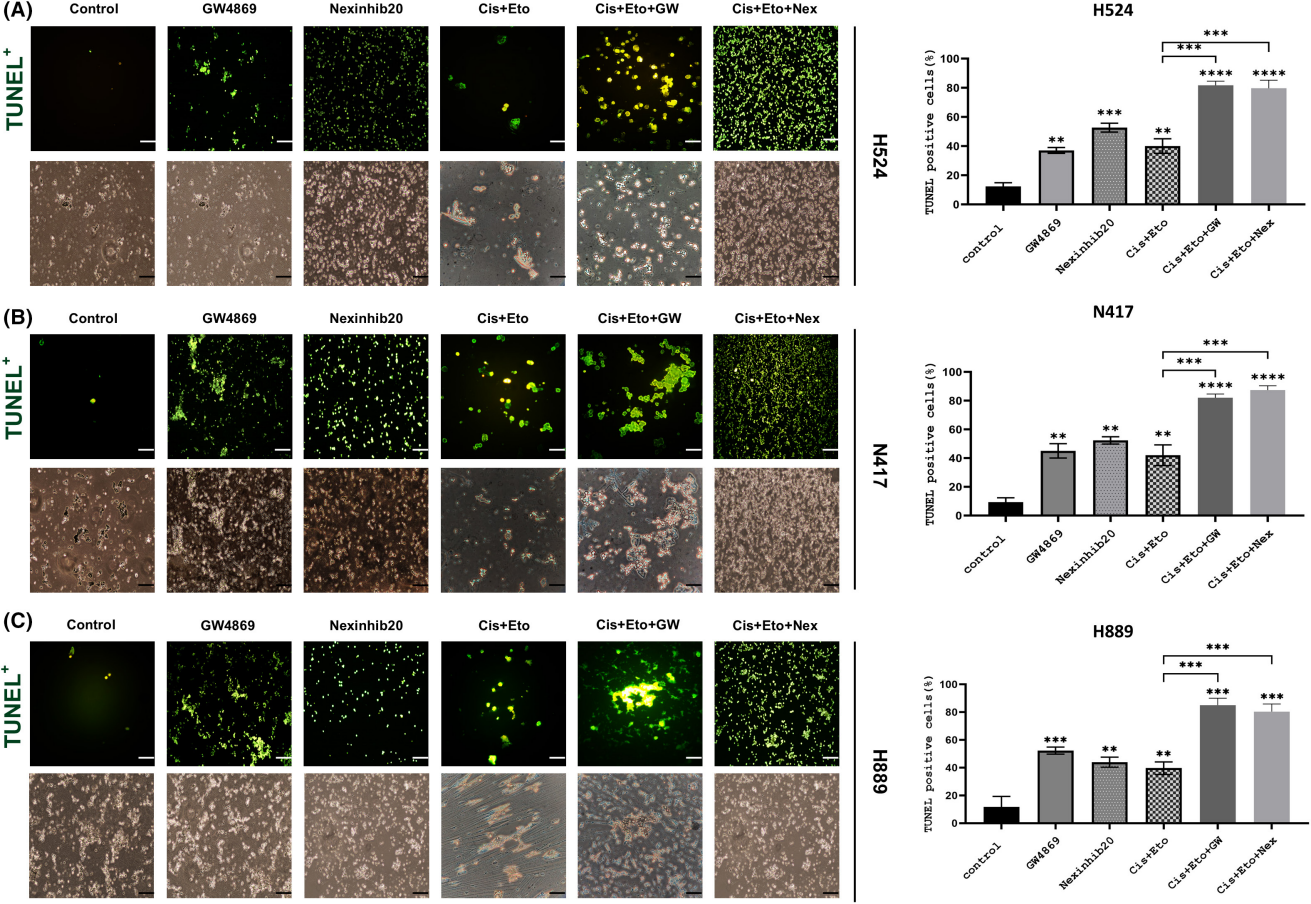
TUNEL assay has shown that inhibition of exosome release and biogenesis induces apoptosis in SCLC cells. Representative pictures (right) and relative quantification graphics of TUNEL‐positive SCLC cells including H524 (A), N417 (B) and H889 (C) treated with IC50 dose of GW4869 or Nexinhib20 in both alone and combination Cis + Eto therapy for 48 h. One‐way ANOVA, *N* = 3. The green colour indicated DNA damage of all apoptotic SCLC cells (original magnification, ×20), and microscopic images of the identified cells accompany the above fluorescence picture.

**FIGURE 7:**
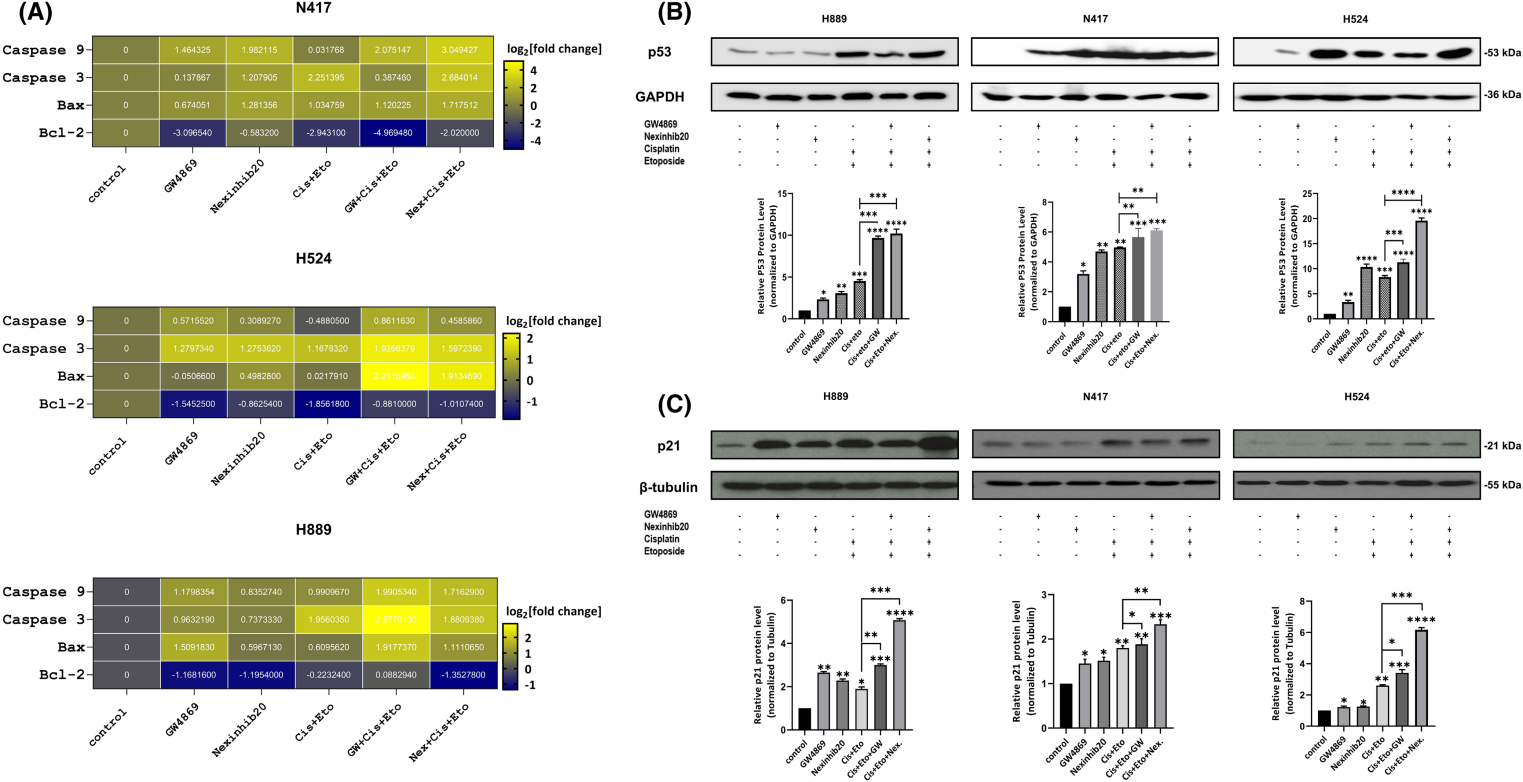
The inhibition of exosome release and biogenesis regulates apoptosis‐related mRNA and protein expressions. Casp9, Casp3, Bax and BCL2 mrna expressions in SCLC cells treated with IC50 dose of GW4869 or Nexinhib20 in both alone and combination Cis + Eto therapy (A), Western blot analysis of protein expression of p53 (B) and p21 (C). Statistical histograms of protein expression determined using western blot and band intensity are assessed quantitatively by LICOR bioscience software. One‐way ANOVA analyzes comparison between all groups and the experiments are repeated three times. *p < 0.05, **p < 0.01, ***p < 0.001, ****p < 0.0001 compared with the non‐treated and cisplatin‐plus etoposide groups of cells.

The combined effects of exosome inhibition on enhancing the efficacy of conventional cisplatin plus etoposide‐based therapy were investigated using the TUNEL assay. N417, H524 and H889 cells were treated with various IC_50_ doses of GW4869 and Nexinhib20 in combination with first‐line treatment, controls and first‐line therapy alone. TUNEL fluorescence labelling showed an increase in the percentage of TUNEL‐positive cells in Cis + Eto + Gw or Cis + Eto + Nex treated H524 (Figure 6A) (*p* < 0.001), N417 (Figure 6B) (*p* < 0.001) and H889 (Figure 6C) (*p* < 0.001) cells compared to Cis + Eto treated cells. qRT‐PCR analysis showed a significant correlation between apoptotic cell index and changes in Bcl2, Bax, Casp3 and Casp9 gene expression (Figure 7A). In addition, after combined treatment of Cis + Eto with GW4869 or Nexinhib20, higher levels of p53 and p21 protein expression were observed in SCLC cell lines compared to controls. (Figure 7B,C). These results suggest that in H889, H524 and N417 cells, Cis + Eto + Gw or Cis + Eto + Nex treatment enhanced the apoptotic effects of Cis + Eto treatment by upregulating p53 and p21.

## 4 DISCUSSION

The primary treatment for SCLC has mainly been unaltered for almost three decades, involving combining a platinum‐based agent (cisplatin or carboplatin) and etoposide (a topoisomerase‐II inhibitor). While most patients with SCLC respond positively to chemotherapy initially, chemo‐resistant carcinomas usually emerge post‐treatment, ultimately leading to relapse and poor survival rates. Combined targeted drug treatments might serve as a novel potential remedy for tumours not receptive to current pharmaceutical therapies. Cancer‐cell‐derived sEV establishes a favourable microenvironment for future metastatic sites, as do primary tumours.^6^, ^7^, ^8^, ^9^ Consequently, the elimination of these cancerous sEVs from the circulatory system has become an innovative and potentially valuable strategy for drug development that prevents the spread of cancer. EVs that circulate in various body fluids, such as cerebrospinal fluid, urine and blood, can be reliable biomarkers of pathophysiological processes. For instance, cancer patients have demonstrated increased EV levels in their blood samples.^24^, ^25^ Furthermore, EVs can also facilitate tumour spread and survival by transporting various miRNAs, soluble proteins and pathological growth factor receptors.^27^, ^28^ The functional proteins or non‐coding RNAs contained in exosomes secreted by tumour cells mediate drug resistance through the regulation of drug efflux and metabolism.^29^ Research suggests that the diversion of EVs from cancer cells contributes to their resistance to chemotherapeutic agents through increased active drug efflux.^22^, ^28^, ^29^, ^30^, ^31^, ^32^, ^33^, ^34^, ^35^, ^36^, ^37^, ^38^, ^39^, ^40^, ^41^, ^42^, ^43^, ^44^, ^45^, ^46^ Recent pharmacological studies have shown that inhibiting EV release could be a new strategy to make cancer cells more susceptible to anticancer drug treatment.^32^, ^37^, ^38^, ^39^ While significant progress has been made in exosome/sEV research in recent years, no medications that specifically target the inhibition of sEV secretion have been approved for human use. This study utilized GW4869 and Nexinhib20 as inhibitors of exosome synthesis and trafficking. GW4869 is a neutral, selective nSMase2 inhibitor, while Nexinhib20 is a potent inhibitor of the small GTPase Rab27a and JFC1 interaction. GW4869 is the most commonly used pharmacological agent to reduce exosome release and inhibit exosome formation. One potential mechanism by which Nexinhib20 inhibits exosome release is through the mediation of pi‐pi stacking interactions between the inhibitor and the critical residue Tyr122 in RAB27A pockets. Therefore, by occupying this crucial residue, Nexinhib20 can potentially disrupt the function of RAB27A and hinder the release of exosomes.^26^

This study provides the first assessment of the inhibitory impacts of GW4869 and Nexinhib20 on SCLC cells by demonstrating that they can restrain the synthesis and trafficking of exosomes. It indicates that GW4869 and Nexinhib20 cause changes in exosome size, leading to decreased expression of CD9, CD63, Tsg101, RAB27A and nsMase2 markers responsible for releasing and trafficking exosomes at both RNA and protein levels. The results also suggest that the combined treatment of exosome inhibitors and first‐line chemotherapy (cisplatin/etoposide) can be an efficient therapy for SCLC.

Our article previously reported that sEVs obtained from these SCLC cells can modify non‐tumorigenic cell homeostasis, promoting their proliferation, invasion and migration in HUVEC and MRC‐5 cells.^13^ Our study demonstrates that GW4869 and Nexinhib20‐mediated exosome reduction reliably leads to decreased cellular proliferation and induces apoptosis. Depending on cellular proliferation and apoptosis results, we analysed the possible expression changes of cell cycle (Cyclin D1 and CDK6) and apoptosis‐related genes with qRT‐PCR and p53 expression changes with western blot analysis. Similarly, the TUNEL assay and qRT‐PCR results showed a significant correlation between apoptotic cell index and Bcl2, Bax, Casp3 and Casp9 gene expressions. Similar to our study, some researchers reported inhibiting exosomes inhibited cell proliferation and promoted apoptosis.^47^, ^48^ In addition, in the literature, Kavanagh et al. showed that treatment with GW4869 increased the expression of p21, which is regulated by p53 in response to a breast cancer model.^49^

This study has shown for the first time that GW4869 and Nexinhib20 could induce apoptosis and repress cellular proliferation by repressing exosome release and biogenesis in human small‐cell lung carcinoma cell lines.

Furthermore, we demonstrate that the co‐treatment of exosome inhibitors with standard chemotherapy (cisplatin/etoposide) strengthens the inhibitory effects on SCLC cells compared to the previously applied standard care, which consists of cisplatin and etoposide.

The primary restriction of the current study is that it was solely conducted in an in vitro setting. Further studies are recommended for a better understanding of the mechanism of the effect of GW4869 and Nexinhib20 on SCLC cell proliferation.

Although numerous in vitro, preclinical in vivo, and clinical studies indicate the ability of various compounds to inhibit the biogenesis and release of exosomes or EVs, additional, comprehensive research with in vitro and in vivo models is necessary to investigate the activities of these compounds for the development of single or combination treatment strategies. At different exosome biogenesis and release stages, these compounds target other proteins. Moreover, the importance of utilizing exosome inhibitors as complementary agents for cancer treatment will increase gradually with the advancement of exosome biology research. Therefore, novel approaches targeting exosomes to attenuate the pathological communication implicated in cancer are likely to have significant therapeutic potential for the future of cancer patients.

We demonstrated the effective reduction of proliferative activity and improved inhibition effects of cisplatin plus etoposide‐based therapy on carcinogenesis by inhibiting exosome release in small‐cell lung cancer cells. Thus, we propose a new potential therapeutic strategy involving the inhibition of exosomes in addition to cisplatin plus etoposide for treating small‐cell lung cancer. In addition, it is advantageous for future in vivo investigations that no pre‐treatment with exosome‐inhibiting therapy was needed to enhance the chemotherapeutic effect.

## AUTHOR CONTRIBUTIONS

**Nesrin Irep:** Conceptualization (equal); data curation (equal); software (equal); validation (equal). **Kubilay Inci:** Conceptualization (equal); data curation (equal); resources (equal); software (equal); visualization (equal); writing – original draft (equal). **Pervin Elvan Tokgun:** Validation (equal); writing – original draft (equal). **Onur Tokgun:** Funding acquisition (equal); supervision (equal); writing – original draft (equal).

## CONFLICT OF INTEREST STATEMENT

All authors declare that they have no conflict of interest.

## Data Availability

The data that support the findings of this study are available from the corresponding author upon reasonable request.
